# Modulation of inhibitory control by prefrontal anodal tDCS: A crossover double-blind sham-controlled fMRI study

**DOI:** 10.1371/journal.pone.0194936

**Published:** 2018-03-28

**Authors:** Etienne Sallard, Michael Mouthon, Michael De Pretto, Lucas Spierer

**Affiliations:** Neurology Unit, Medicine Department, Faculty of Sciences, University of Fribourg, Fribourg, Switzerland; University Medical Center Goettingen, GERMANY

## Abstract

Prefrontal anodal transcranial direct current stimulation (tDCS) has been proposed as a potential approach to improve inhibitory control performance. The functional consequences of tDCS during inhibition tasks remain, however, largely unresolved. We addressed this question by analyzing functional magnetic resonance imaging (fMRI) recorded while participants completed a Go/NoGo task after right-lateralized prefrontal anodal tDCS with a crossover, sham-controlled, double-blind experimental design. We replicated previous evidence for an absence of offline effect of anodal stimulation on Go/NoGo performance. The fMRI results revealed a larger increase in right ventrolateral prefrontal activity for Go than NoGo trials in the anodal than sham condition. This pattern suggests that tDCS-induced increases in cortical excitability have larger effects on fMRI activity in regions with a lower task-related engagement. This was the case for the right prefrontal cortex in the Go condition in our task because while reactive inhibition was not engaged during execution trials, the unpredictability of the demand for inhibitory control still incited an engagement of proactive inhibition. Exploratory analyses further revealed that right prefrontal stimulation interacted with task-related functional demands in the supplementary motor area and the thalamus. Our collective results emphasize the dependency of offline tDCS functional effects on the task-related engagement of the stimulated areas and suggest that this factor might partly account for the discrepancies in the functional effects of tDCS observed in previous studies.

## Introduction

Inhibitory control refers to the ability to suppress irrelevant cognitive or motor responses [1]. Current functional literature indicates that motor inhibitory control is supported by right inferior frontal gyrus (rIFG) and pre-supplementary motor areas (preSMA), which suppress thalamocortical motor programs via their projections to the subthalamic nuclei (e.g. [2–4]). In line with these findings, transcranial direct current stimulation (tDCS) studies report that modulations of ventrolateral prefrontal cortex (VLPFC) excitability can improve inhibition performance [5–12]. Jacobson and colleagues [7] for instance report shorter stop signal reaction times (SSRT) after excitatory anodal than sham tDCS over the rIFG during a stop-signal task (SST; see also [10, 11]; and [6, 8, 12] for similar results when applying anodal tDCS over the preSMA).

Although all VLPFC tDCS studies based on SST report performance improvements after excitatory stimulation, such effects were not observed when using Go/NoGo tasks [13–17]. For example, Beeli and colleagues [15] applied tDCS over the right dorsolateral prefrontal cortex (DLPFC) before a Go/NoGo task and found no behavioral effect after anodal tDCS and a decrease of performance (as indexed by an increase of false alarm rate) after cathodal tDCS. In a recent study applying tDCS over the rIFG, Campanella et al. [16] found no behavioral effect of anodal stimulation during a Go/NoGo task. Finally, Cunillera et al. [17] used a hybrid inhibition task with Go, NoGo and GoStop-trials, and found a longer response time in the Go condition during anodal compared to sham rIFG stimulation, as well as inhibitory control improvements (decreased Stop-Signal Reaction time).

The findings for improvements in SST but not Go/NoGo performance with anodal stimulation could have resulted from a difference in the demand for inhibition between the two tasks. Since SSTs require inhibiting ongoing motor responses and Go/NoGo tasks only prepotent responses, the demand for reactive inhibition is stronger in SST than in Go/NoGo tasks [18–20]. Reactive inhibition may thus benefit more from an enhanced proactive inhibition in SST than in Go/NoGo tasks. Since rVLPFC tDCS has been shown to enhance both proactive and reactive inhibition [9, 21], it should have larger effects on SSTs than on Go/NoGo tasks. Critically, this hypothesis predicts an increase in rVLPFC activity during both Go and NoGo trials in Go/NoGo tasks after a-tDCS, because an increase in excitability may potentiate the proactive engagement of this region during both types of trials.

However, to our knowledge, no study tested this latter prediction, nor more generally how PFC tDCS influences inhibition-related functional activity. Only three electroencephalography studies (EEG; [13, 16, 17]) and one functional magnetic resonance imaging study examined the effect of tDCS on inhibitory control (fMRI; [12]). After applying tDCS over the rIFG, Cunillera et al. [17] and Campanella et al. [16] observed a decrease in P3 amplitude during NoGo and/or stop trials in anodal compared to sham stimulation, whereas left M1 tDCS did not influence electrophysiological activity during a subsequent Go/NoGo task in Conley et al. [13]. Decreases in P3 amplitude during response inhibition were generally interpreted as reflecting the need for the recruitment of less neural resources after anodal stimulation. The fMRI study by Yu et al. [12] used tDCS over the preSMA during an SST and reported increased activity in the preSMA after anodal stimulation during stop trials, associated with improved inhibitory control (i.e. decreased SSRT), suggesting that higher fMRI activity was related to faster stopping speed.

Current studies on PFC tDCS during inhibitory control tasks thus measured only the behavioral effects of the stimulation [5–11, 15] or used functional methods with limited neurophysiological interpretability [16, 17, 22], leaving unclear the functional effects mediating tDCS-induced changes in inhibitory control performance. Moreover, most of these studies did not use a double-blind approach and were thus potentially confounded by participants’ or experimenters’ expectations [23]. The present study overcomes these limitations by using a double-blind, sham-controlled crossover design to investigate the behavioral and functional offline effects of right-lateralized PFC anodal tDCS on inhibitory control with fMRI. We used a Go/NoGo task to clearly differentiate between the effects of the stimulation on execution and inhibition trials. We hypothesize that while the anodal stimulation may not necessarily improve subsequent inhibition performance, it should increase the activity of the stimulated inhibition areas during the execution trial and not forcibly during inhibition trials (a change in excitability may indeed not manifest on task-related activity of strongly recruited areas [24–26]). Although our primary focus was on rVLPFC activity, we adopted a whole-brain approach to detect potential effects of the tDCS beyond this area of interest.

## Material and methods

### Participants

Nineteen right-handed [27] healthy adults participated to this study. None of the participants reported history of neurological and psychiatric disease. Three participants were excluded due to head movements during the scanning, technical problems, or mean reaction time (RT) and missed responses in the Go/NoGo task greater than 2.5 standard deviations from the group mean. A total of 16 participants were thus eventually included in the analyses (9 females; mean age ± standard error: 23.8 years ± 3.9; range: 18–36).

### Ethical statement

The research protocol was approved by our local ethics committee (Commission cantonale (VD) d'éthique de la recherche sur l'être humain, CER-VD 239/14). All procedures followed were in accordance with the ethical standards of the responsible committee on human experimentation (institutional and national) and with the Helsinki Declaration of 1975, as revised in 2008. Each participant provided written informed consent to the study.

### Procedure and task

The experiment consisted of two sessions separated by at least 7 days (mean: 8; range: 7–12 days). Each session began with 20 min of tDCS stimulation (either anodal or sham stimulation) immediately followed by the Go/NoGo task in the fMRI scanner. The participants and the experimenters were blind to the tDCS condition (pseudo-randomized counterbalanced order of the anodal or sham stimulation). The average delay between the end of the stimulation and the beginning of the MRI Go/NoGo task for 15 participants (one missing value) was 9.2 minute in anodal and 9.1 minute in sham stimulation; there was no difference between the delay in the anodal vs. sham stimulation (p = 0.47).

### Go/NoGo task

The Go/NoGo task was the same as in Chavan et al. [28]. It consisted of nine different white letters (A, E, H, I, M, O, S, T, X) presented in the center of a black screen. NoGo stimuli were the letters X; Go stimuli were the remaining letters. A fixation cross (1200–2200 ms) was presented before one of the nine letters (500 ms). Participants had up to 1700 ms to respond.

Participants had to respond as fast as possible to Go stimuli by pressing a button with the right index finger and to withhold their responses to NoGo stimuli. To build up a tendency to respond and thereby increasing the inhibitory effort necessary to successfully withhold the responses to NoGo stimuli, the task was weighted towards Go stimuli (Go trial: 70%; NoGo trial: 30%). A total of 5 blocks of 80 trials were performed in each session. Each block consisted of 56 Go and 24 NoGo trials presented randomly.

After the Go/NoGo paradigm and within the same fMRI acquisition session, a supplementary control task was performed to isolate brain activation related to motor action (tapping condition). A press-symbol was presented with the same timing as the Go/NoGo task. Participants were required to press on the button each time they saw a press-symbol.

Stimulus delivery and response recording were controlled using E-Prime 2.0. The total duration of the fMRI run was of 20 minutes. The total duration of the MRI session was about 35 minutes.

### Transcranial direct current stimulation

Transcranial DCS was delivered by a battery-driven, constant current stimulator (DC-Stimulator Plus, www.neuroconn.de). A direct current of 1.5mA for 20 min was induced by two sponge-electrodes (anode: 5 x 7 cm; cathode: 7 x 10 cm) soaked in saline solution. Anodal and sham stimulation had the same electrode configuration, with the target electrode over rIFG and the reference electrode over the left orbito-frontal cortex (lOFC). The target electrode (anodal in the real stimulation condition) was placed over F6-FC6 (using the 10–20 EEG convention) and the reference electrode (Cathode in the ‘real’ stimulation condition) was positioned above the left eyebrow [29]. A ramp up/ramp down period of 30/15 seconds was applied at the beginning and end of the tDCS session. Sham stimulation consisted in the same fade-in/out but with the stimulation set back to zero during the 20 minutes. This way, the sensation of the stimulation was identical between the anodal and sham stimulation (confirmed by the post-session evaluation of the tDCS side-effects (all p-values >0.05)).

**Fig 1:**
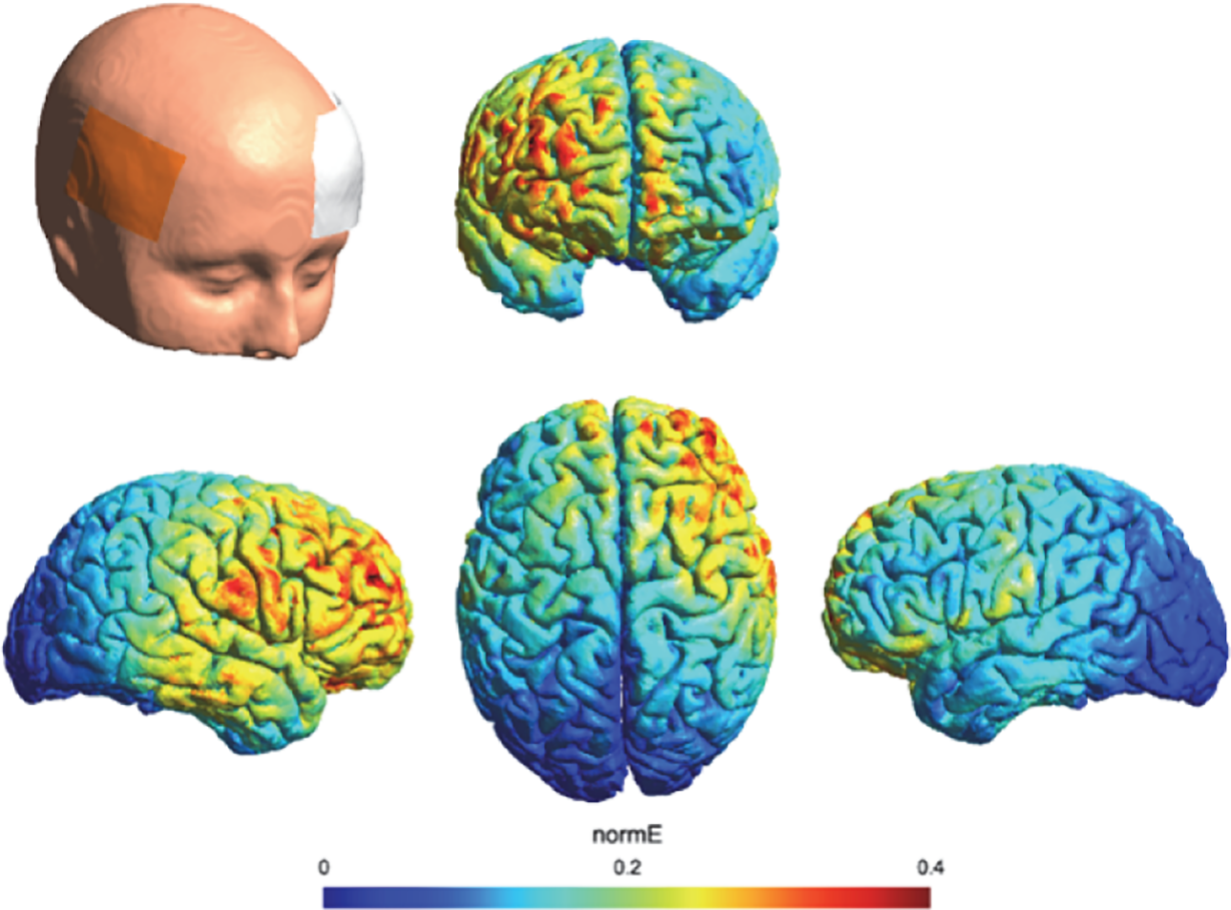
tDCS electric field distribution estimate. Results of the electric field distribution estimate are shown for the anode electrode over the rIFG (F6-FC6) and the cathode electrode over the left OFC. The electric field strength is scaled from 0 (minimum: blue) to 0.4 mV/mm (maximum: red).

We report in Fig 1 an estimate of the electric field induced by our tDCS montage in the anodal condition. The estimation of the distribution of the electric field was generated in SimNIBS 2.0.1 [30]. The model is based on the following conductivity values for its key anatomical components (SimNIBS default values, as in e.g. [30, 31]): scalp (σ = 0.465 S/m), bone (σ = 0.010 S/m), cerebrospinal fluid (σ = 1.654 S/m), gray matter (σ = 0.275 S/m), and white matter (σ = 0.126 S/m). The volume mesh and visualization were generated through Gmsh [32].

### Behavioral analyses

The mean RT in Go (RT HIT) and the rate of commission error in NoGo (False Alarm: FA) were compared between the stimulation conditions (anodal vs. sham) with paired t-tests. An efficiency index [33, 34] calculated as the mean RT Hit divided by the percentage of Correct Reject NoGo (100—FA rate) was also computed for each participant in each session to have a single-value measure of performance taking into account both speed and accuracy. The same analyses for the effect of the sessions (session 1 vs. session 2) are reported in the supplementary material (S1 Fig and S1 Table).

### Functional magnetic resonance imaging (MRI) recording

Neuroimaging data were recorded with a 3T MRI scanner (Discovery MR750; GE Healthcare, Waukesha, Wisconsin), equipped with a 32-channel head coil. To reduce head movements, the head of the participants was stabilized with a sound-attenuating memory foam. Stimuli were presented on a LCD screen at 60Hz (NordicNeuroLab, Bergen, Norway) with visual angles of 1.34° (height) by 1.2° (length).

T1-weighted images were acquired with a FSPGR BRAVO sequence (voxel size: 0.86 x 0.86 x 1 mm, number of coronal slices: 276, TR/TE = 7300/2.8 ms, prep time = 900 ms, flip angle = 9°, parallel imaging acceleration factor (PIAF):1.5, intensity correction (SCIC)).

Functional T2*weighted echo planar images with blood oxygenation level-dependent (BOLD) contrast were acquired with the following parameters: voxel size: 2.3 x 2.3 x 3 mm, 37 ascending axial slices, inter-slice spacing = 0.2 mm, TR/ TE = 2000/30 ms, flip angle = 85°, PIAF: 2. Each session was preceded by 8 seconds of dummy scans to ensure a steady-state magnetization of the tissues. A total of 601 volumes were acquired during this acquisition.

In order to correct for the distortions due to inhomogeneity of the magnetic field, two FAST SPGR with gradient echo sequences were acquired with distinct Echo Time at the beginning of the MRI session: TE1/TE2 = 4.9/7.3 ms, TR = 50 ms, filp angle = 45°, same spatial coverage as the T2* weighted acquisition.

### MRI analyses

MRI data were analyzed using the SPM12 software (the Welcome Trust Centre for Neuroimaging, Institute of Neurology, University College London). The T1-weighted images of both sessions were coregistered to compute their mean. Functional MRI images were preprocessed following a standard procedures [35] including: realignment and unwarping using the fieldmap of each sessions, realignment of the second session on the first session, slice timing, spatial normalization to the Montreal Neurological Institute (MNI) space with 3x3x3 mm^3^ voxel size using the improved version of the “unified segmentation” [36, 37] on the mean T1-weighted coregistered on fMRI images native space, and finally a smoothing with an isotropic 8-mm full width at half-maximum (FWHM) Gaussian kernel. The Artrepair toolbox was used to detect the presence of rapid movements between the fMRI images (one subject was excluded because more than 10% of scans showed rapid motion above 0.5 mm/TR for both sessions).

The preprocessed fMRI images were submitted to fixed effects analyses at the subject level by applying a general linear model to each voxel [38]. For the Go/NoGo task, each stimulus onsets were modeled as a delta function and convolved with the hemodynamic response function in an event-related strategy of analysis. Only the correct Go (RT HIT) and NoGo (correct rejections) were considered in the analysis (misses and false alarms were modeled as conditions of no interest). The trials of the tapping task were integrated in the model as a block. In addition, movement parameters were included as regressors of no-interest. Time series from each voxel were high-pass filtered with a 1/250 Hz threshold to remove low frequency noise and signal drifts. In addition, an auto-regressive function (AR(1)) was applied to correct for temporal correlations between neighboring voxels.

Since there was a manual response in the Go but not in the NoGo condition, we subtracted the activation during the tapping block from the responses to the Go trials by contrasting them (Go > tapping) to prevent motor activity from the button press to contaminate the results. This contrast, as well as the simple NoGo vs. baseline contrast, were computed as fixed effect analyses and then submitted to 2*2 flexible factorial model with repeated measures (Condition (NoGo; Go) * Stimulation (anodal; sham)) at the second level analyses. In this random effect analysis, the main effects of factors Stimulation (anodal vs. sham) as well as the interaction between experimental factors were analyzed in both directions by t-contrasts on the whole brain. The significance threshold for the functional results was set at p<0.05 FWE corrected for multiple comparisons at the voxel level with a minimal cluster size of three contiguous voxels. The anatomical position of clusters’ maxima was localized in the MNI space with the Neuromorphometrics probabilistic atlas available in SPM12 [39, 40]. The results are displayed according to the neurological convention. We further report as supplementary material the results of the interaction with a p<0.05 cluster-wise correction threshold (p_uncor_<0.001, k = 100) to facilitate the comparison of our result with previous functional literature using this correction approach (S2 Fig and S2 Table).

## Results

### Behavior

**Fig 2:**
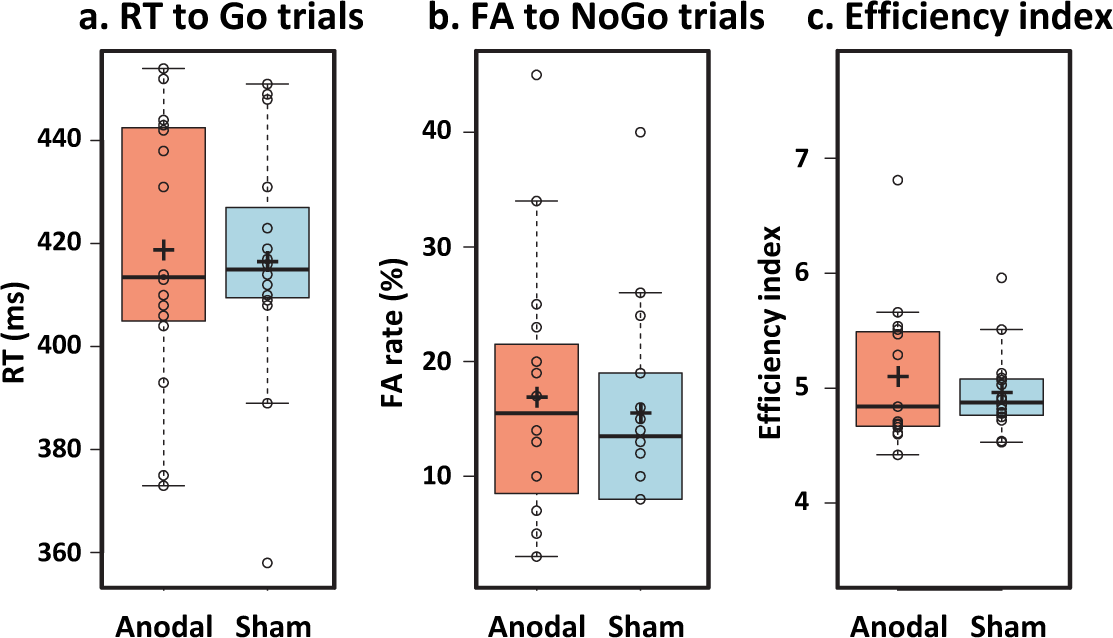
Behavioral results: Anodal vs. Sham stimulation. Box plots representing the RT HIT, FA rate and efficiency index in anodal and sham stimulation (a, b and c). Individual subject’s data points, the median (horizontal line), the mean (cross), and confidence intervals (Tukey whiskers) are represented.

There was no difference between the anodal vs. sham stimulation on the response time to Go trials (Fig 2A and Table 1; boxplots generated with the BoxPlotR webtool by Spitzer et al. [41]), nor on the FA rate to the NoGo trials (Fig 2B and Table 1). The same analysis applied to the inverse efficiency index (EI) showed no difference in the performance between the anodal and sham stimulation when taking into account both speed and accuracy in a single value index (Fig 2C and Table 1).

**Table 1:** Behavioral results. Anodal vs. sham stimulation.

STIMULATION	RT HIT	FA rate	Efficiency Index
**ANODAL**	419±26	17±11	5.1±0.6
**SHAM**	416±23	16±9	4.9±0.4
**P-value**	0.60	0.55	0.30
**T-value**	0.53	0.61	1.06
**Cohen’s d**	0.13	0.15	0.27
**Bayes factor (B01)**	3.44	3.40	2.49

Response time to Go condition (RT-HIT), false alarm rate (FA) and efficiency index in the anodal and sham conditions; means ± standard deviations are represented, p- and t-values comparison (paired t-test), Cohen’s d effect size and Bayes factor.

The results of the comparison between the first and the second session (independent of the stimulation type) are reported in supplementary materials (S1 Fig and S1 Table).

Given the limitation of the frequentist approach to provide support for the null hypothesis, we conducted Bayes factors analyses using the free software JASP (JASP Team, 2018, https://jasp-stats.org/), with the default Cauchy prior width (r = .707). Bayes factors express the probability of the data given H0 relative to H1 (i.e, substantial evidence for the null or alternative will be considered as Bayes factors of >3 or <0.33 respectively [42]). The BF01 for the RT and FA were around 3 (Table 1), indicating substantial support for the null (the data were ca. 3 times more likely observed under the null hypothesis).

### Functional MRI

The functional activity during the post-tDCS Go/NoGo task was analyzed with a 2*2 flexible factorial repeated measures ANOVA with the factors Condition (NoGo; Go) and Stimulation (anodal; sham). A stringent alpha threshold of p_FWE_<0.05 corrected at the voxel level was chosen for the whole brain functional analyses (see supplementary material: S2 Fig for the p-map with a p<0.05 cluster-wise correction threshold (p_uncor_<0.001, k = 100)).

**Fig 3:**
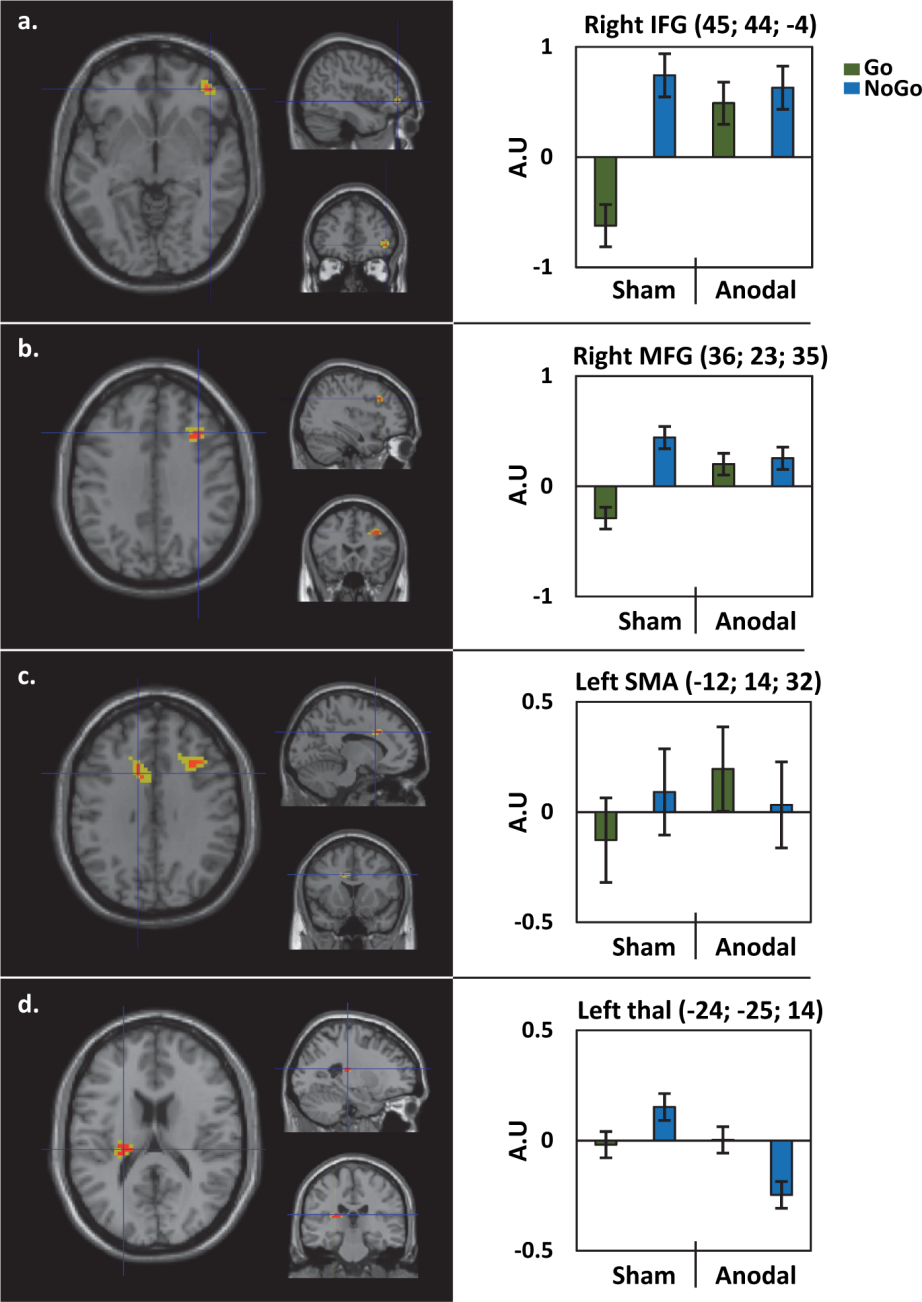
Functional neuroimaging results: Stimulation (Anodal; Sham) by Condition (Go; NoGo) Interaction. Results are shown on a normalized single-subject brain in the MNI space. Contrasts are represented at p_FWE_< 0.05 corrected at voxel level (min cluster size = 3, red) and to facilitate visualization p_FWE_< 0.3 in yellow). Histograms on the right indicate the amplitude of the effect (Arbitrary Unit: A.U) for each condition at clusters’ local maxima with standard error bars. MFG = middle frontal gyrus, IFG = inferior frontal gyrus, SMA = supplementary motor area (see supplementary materials for statistical details).

There was a significant interaction ((NoGo-Go)_sham_>(NoGo-Go)_anodal_) within the right inferior frontal gyrus (rIFG), the right middle frontal gyrus (rMFG), the left supplementary motor area (lSMA) and the left thalamus (Fig 3 and Table 2).

**Table 2:** Locations of clusters local maxima for the functional MRI interaction.

Anatomical region	Vx	MNI Coordinates			Z
Left thalamus proper	14	-24	-25	14	5.10
Right middle frontal gyrus	12	36	23	35	4.99
Right inferior frontal (par triangularis)/middle frontal	3	45	44	-4	4.72
Left supplementary motor cortex (SMA)*	4	-12	14	32	4.69

Stereotaxic brain MNI coordinates for peak-voxels of the functional interaction (NoGo-Go)_sham_>(NoGo-Go)_stim_. Results are represented at p_FWE_< 0.05 corrected at the voxel level (min cluster size = 3). Vx = voxel, Z = Z-score of the peak.

In the frontal clusters (i.e. rIFG and rMFG), the interaction was driven by lower activity in the Go than NoGo condition after sham but not anodal stimulation (Fig 3A and 3B).

In the lSMA (Fig 3C), the interaction was driven by higher activity in the Go than NoGo after anodal stimulation, with the reverse pattern after sham stimulation.

In the left thalamus (Fig 3D) the interaction was driven by lower activity in the NoGo anodal than NoGo sham stimulation, with no effect of stimulation on the Go condition.

Of note, together with the behavioral results for ca. 15% FA and 400ms RT, the fact that the Go vs. NoGo functional MRI contrast in the sham condition replicated the typical pattern for a larger involvement of the right VLPFC during motor response inhibition [4, 43] confirms that our task adequately engaged inhibition processes and that our results could be generalized to classical Go/NoGo tasks (see S3 Fig).

## Discussion

We identified the behavioral and functional effects of right-lateralized PFC tDCS during a subsequent Go/NoGo task using fMRI. Our double-blind sham-controlled crossover design ensured that participants’ or experimenters’ expectations on the effects of the tDCS did not confound the results. We replicated previous observations for an absence of after-effect of anodal tDCS on Go/NoGo performance. The functional results revealed a larger increase in rVLPFC activity in the Go than NoGo condition after anodal than sham stimulation. Exploratory analyses further revealed differential effects of the anodal stimulation on Go vs NoGo trials beyond the rVLPFC area, on the SMA and the thalamus.

### Anodal tDCS increases rVLPFC during Go but not NoGo trials

The results of the Stimulation by Condition interaction revealed a larger increase in rVLPFC activity in the anodal than sham condition for the Go than NoGo condition. In line with previous functional literature on motor inhibitory control (e.g. [4]), our results indeed indicate more rVLPFC activity in the NoGo than Go trials in the baseline (sham) stimulation condition. The larger effect of tDCS during Go than NoGo trials thus likely resulted from a lower recruitment of the rVLPFC during the execution than inhibition trials.

Although in the Go sham condition the BOLD signal in the rVLPFC cluster was low, this area was likely involved during the execution trials. In experimental contexts where forthcoming demands for response inhibition cannot be predicted as in our reactive Go/NoGo task, proactive inhibition is indeed strategically engaged before all trials to reduce false alarms rate. Inhibition should however not be too much pre- engaged because it would increase response time to Go trials and thus conflict with the instruction to respond as fast as possible. In our task, speed-accuracy trade-off optimizations thus called for the engagement of a weak proactive inhibition and in turn induced a weak activity in the rVLPFC during Go trials. An interaction between the proactive engagement of the VLPFC during execution trials and the increase in excitability of this area via a reductions of GABA levels by the anodal tDCS thus possibly explains the observed increase in fMRI activity [29, 44].

Our finding for different effects of tDCS depending on the task-related engagement of the stimulated areas echoes previous reports for interactions between tDCS and the subjects’ neurocognitive states [45–48]. Interactions between tDCS and functional activity were however observed in various directions, with for example anodal tDCS being associated with a reduction in BOLD signal in some studies [24, 25], but with an increase in others ([26]; see [49, 50] for discussion). These discrepancies even led some authors to question whether tDCS actually has any neurophysiological effects beyond changes in motor-evoked potentials [51]. In this regard, our pattern of results suggests that the baseline level of engagement of the brain areas of interest might be a key factor in determining the functional effect of tDCS, with changes in BOLD signal after anodal tDCS manifesting only under conditions of low task-related activity. A better control of this aspect could thus help improving the reliability of the effects of tDCS on brain activity, and by extension on its behavioral consequences.

Previous findings for improvements in SST but not Go/NoGo performance with anodal rPFC stimulation could likewise be accounted for by interactions between tasks demands and the tDCS. Since SSTs require inhibiting ongoing motor responses and Go/NoGo tasks only a prepotent response, the demand for reactive inhibition is stronger in SST than Go/NoGo tasks. Reactive inhibition may thus benefit more from an enhanced proactive inhibition in SST than Go/NoGo tasks. Since rVLPFC tDCS has been shown to enhance both proactive and reactive inhibition [9], it should have larger effect on SST than on Go/NoGo task. In that sense, our finding for an increase in VLPFC activity during the Go trials confirms that an effect of the stimulation on proactive inhibition possibly accounts for the task-specificity of the effect of tDCS on inhibitory control.

### Right PFC anodal tDCS does not improve subsequent Go/NoGo performance

The lack of offline effect of tDCS on Go/NoGo performance observed in the present study corroborates the findings of previous Go/NoGo tDCS studies with tDCS on the rIFG [16, 17], the right DLPFC [15] or M1 [13, 14]. The only studies having found an improvement of inhibition accuracy after tDCS used SST and applied tDCS over the rIFG [5, 7, 9–11], preSMA [6, 8, 12, 52] or M1 [52].

While the lack of effect of the tDCS on NoGo-related fMRI activity fits with the absence of change in FA rate after anodal stimulation, it was surprising that the observed increase in VLPFC activity during the Go trials did not result in any change in Go response time. The thalamocortical inputs to motor cortex representation controlling movements during action execution and inhibition are respectively modulated by the premotor cortex-putamen projections and the hyperdirect IFG-subthalamic nucleus pathway (e.g. [53, 54]). The inhibitory activity of the VLPFC during execution trial induced by the tDCS could thus have increased the response times [9]. Our negative result at this level possibly followed from a floor effect: because our task was very simple (easily discriminable Go and NoGo stimuli and simple motor response), the possibility for the tDCS to further decrease the RTs might have been limited [13–16]. We explored this possibility by extracting the Tau parameter of an ex-Gaussian fitting distribution on the RT data using the ExGauss toolbox implemented in Matlab by Zandbelt and Bram (2014; https://github.com/bramzandbelt/exgauss). Since the Tau exponential parameter mainly indexes the right tail of the RT distribution, it might be less influenced floor effects. In addition, the Tau ex-gaussian component has been advanced to index our cognitive process of interest including e.g. inhibitory control [55], or increased task conflict [56, 57]. The comparison of the Tau component between the anodal and sham condition revealed no difference at this level (p = 0.5) and a Bayes factor analysis supported the null hypothesis (BF01 = 3.38), confirming that the tDCS-induced change in rVLPFC activity during the Go trial had no effect on response speed even when focusing on measures less sensitive to potential floor effects.

### Right PFC anodal tDCS has functional offline effect beyond the rVLPFC

Exploratory analyses of the tDCS offline effect outside of the rVLPFC revealed a Stimulation by Condition interaction in the left SMA with higher activity in Go compared to NoGo trials after anodal stimulation. Activity in the SMA has been reported during externally generated movement [58], especially following visual cues [59], and plays a key role in motor preparation [60, 61]. Since SMA is directly connected to M1 [62], the modulation of the left SMA by tDCS might follow from its involvement in the control of the contralateral right hand, which was used by the participants during the task. The generally higher preparatory excitation of this area during the task may have resulted in increase in fMRI activity when coupled with tDCS-modulated inputs from the rIFG and/or with its tDCS (the electric field estimate indeed revealed that our tDCS montage resulted in a stimulation of the SMA). Furthermore, a reduced movement preparation, reflected by low level of SMA activity during the NoGo trials, would have resulted in more efficient inhibition.

Finally, we observed a modulation of the left thalamus by the tDCS, with lower activity in NoGo after anodal than sham stimulation, without change during the Go condition. The thalamus, along with the basal ganglia, plays a regulatory role of motor action through striato-thalamo-cortical loops [63]. An over-excitation of the thalamo-cortical pathways through dysfunction of the striatum may for example result in reduced inhibitory control [64]. Thus, we hypothesis that the increased excitability of the rIFG after anodal tDCS indirectly reduced the need for thalamic regulation during NoGo trials. This explanation would account for the absence of difference in activity between the sham and the anodal conditions: to successfully inhibit motor responses, a certain amount of activation is necessary, whereas over-activation may reduce inhibition performance [65].

### Limitations

Our study suffers several limitations. First, the low temporal resolution of fMRI does not allow differentiating the effect of the tDCS during the sequence of execution and inhibition processes following stimulus onset. Such information could have helped refining our interpretation on the effect of tDCS on proactive vs. reactive inhibitory control. Second, we did not examine whether the online vs. offline tDCS would result in different effect on inhibitory control performance and the associated functional activity. Previous literature on the effects of tDCS on inhibitory control used both online and offline protocols, and difference at this level does not seem to account for the finding that tDCS influences SST but not Go/NoGo performance. For example, offline stimulation improved inhibitory control during SST [9] but not Go/NoGo [16]. Further studies are required to assess whether online stimulation might result in larger effect size on Go/NoGo performance.

Finally, our electrode montage resulted in the stimulation of the right anterior and dorsal PFC as well as in left medial anterior PFC areas that might have influenced our patterns of results. Isolating the influence of changes in the excitability of the rIFG would require additional investigations with more focal brain-stimulation methods such as High Definition tDCS or transcranial magnetic stimulation.

### Conclusions

Our collective results support previous evidence for an absence of effect of right PFC anodal tDCS on Go/NoGo performance and revealed smaller functional effects of the stimulation on inhibition than execution trials within the key inhibition areas. They further suggest that interactions between tDCS and task-related functional activity in the stimulated areas could account for previous observations of variations in the behavioral effect of tDCS across inhibition tasks, and stress that this factor should be considered when generating predictions on the effects of tDCS.

## Supporting information

S1 Fig Behavioral results: Session 1 vs. Session 2 comparison. Box plots representing the RT HIT, FA rate and efficiency index in session 1 and session 2 (a, b and c). Individual subject’s data points, the median (horizontal line), the mean (cross), and confidence intervals (Tukey whiskers) are represented.
(TIF)

S2 Fig Functional magnetic resonance imaging: Cluster-wise corrected Interaction results. Same illustration as the Fig 2 of the article (Stimulation by Condition Interaction), but with an uncorrected statistic at the voxel level (p<0.001) with FWE corrected of the cluster-wise statistic (p<0.05, min size = 100 voxels). There are two distinct clusters showing the interaction (a and b). The results are represented at the same MNI coordinates as the voxel-wise corrected results. Results are shown on a normalized single-subject brain in the MNI space. Histograms on the right indicate the amplitude of the effect (Arbitrary Unit: A.U) for each condition at clusters’ local maxima with standard error bars. MFG = middle frontal gyrus, IFG = inferior frontal gyrus, SMA = supplementary motor area.
(TIF)

S3 Fig Functional magnetic resonance imaging: Main effect results. Functional neuroimaging results for the main effect of the factor Condition (Go; NoGo) calculated as the [CR-(HIT-Tap)] contrast. Results are shown on a normalized single-subject brain in the MNI space. Contrasts are represented at pFWE< 0.05 corrected at voxel level (min cluster size = 3). Regions showing a main effect of conditions include the bilateral inferior occipital gyrus, right superior marginal gyrus, right angular gyrus, and right middle frontal gyrus.
(TIF)

S1 Table Behavioral results for the Session 1 vs. Session 2 contrast. Response time to Go condition (RT HIT), false alarm rate (FA) and efficiency index in session 1 and session 2. We report the means ± standard deviation, p- and t-values of the comparison (paired t-test) and Cohen’s d effect size.
(TIF)

S2 Table Locations of clusters Local maxima for the functional MRI interaction. Stereotaxic brain MNI coordinates for peak-voxels of the functional interaction (NoGo-Go)sham>(NoGo-Go)stim. Results are represented at pFWE< 0.05 corrected at voxel level (min cluster size = 3). An asterisk (*) indicates the anatomical position is retrieved from the nearest GM for this particular peak. Vx = voxel size, Z = Z-score of the peak.
(TIF)
