# Supplementary figures and images for: Modulation of inhibitory control by prefrontal anodal tDCS: A crossover double-blind sham-controlled fMRI study
Source: PLoS One. 2018 Mar 28;13(3):e0194936. doi: 10.1371/journal.pone.0194936 (PMC5874055; doi:10.1371/journal.pone.0194936)

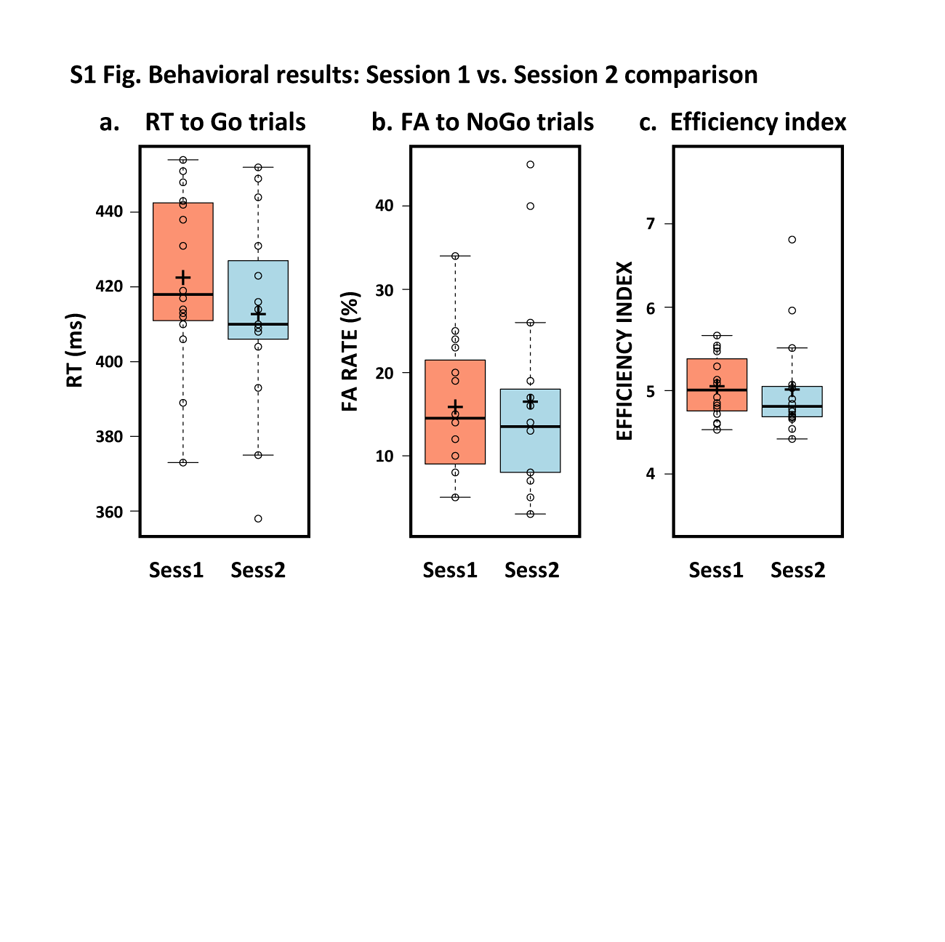

Supplement: S1 Fig — Box plots representing the RT HIT, FA rate and efficiency index in session 1 and session 2 (a, b and c). Individual subject’s data points, the median (horizontal line), the mean (cross), and confidence intervals (Tukey whiskers) are represented. (TIF) [file pone.0194936.s001.tif]

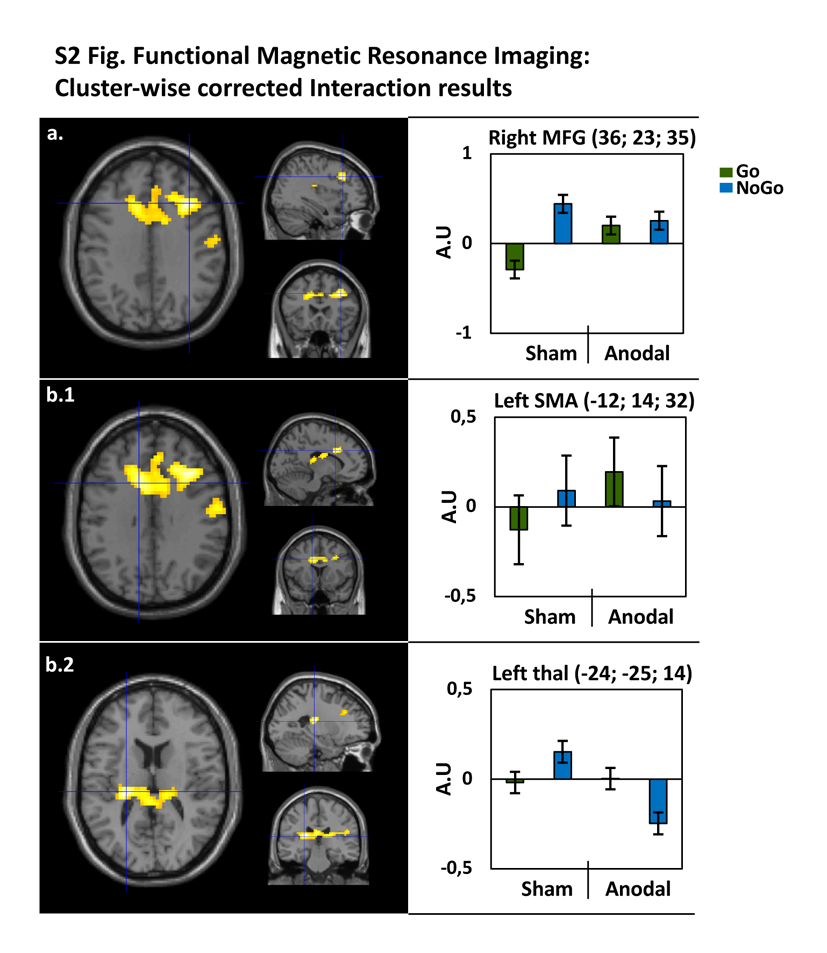

Supplement: S2 Fig — Same illustration as the Fig 2 of the article (Stimulation by Condition Interaction), but with an uncorrected statistic at the voxel level (p<0.001) with FWE corrected of the cluster-wise statistic (p<0.05, min size = 100 voxels). There are two distinct clusters showing the interaction (a and b). The results are represented at the same MNI coordinates as the voxel-wise corrected results. Results are shown on a normalized single-subject brain in the MNI space. Histograms on the right indicate the amplitude of the effect (Arbitrary Unit: A.U) for each condition at clusters’ local maxima with standard error bars. MFG = middle frontal gyrus, IFG = inferior frontal gyrus, SMA = supplementary motor area. (TIF) [file pone.0194936.s002.tif]

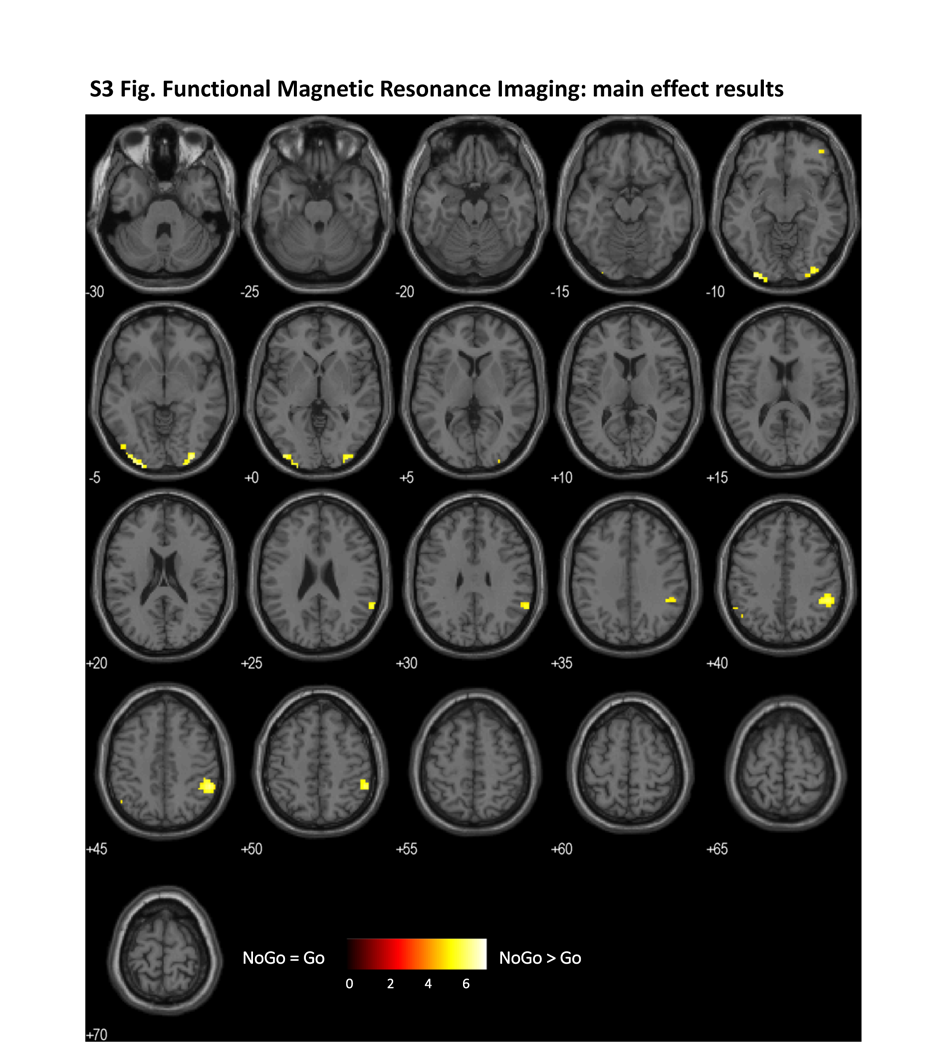

Supplement: S3 Fig — Functional neuroimaging results for the main effect of the factor Condition (Go; NoGo) calculated as the [CR-(HIT-Tap)] contrast. Results are shown on a normalized single-subject brain in the MNI space. Contrasts are represented at pFWE< 0.05 corrected at voxel level (min cluster size = 3). Regions showing a main effect of conditions include the bilateral inferior occipital gyrus, right superior marginal gyrus, right angular gyrus, and right middle frontal gyrus. (TIF) [file pone.0194936.s003.tif]

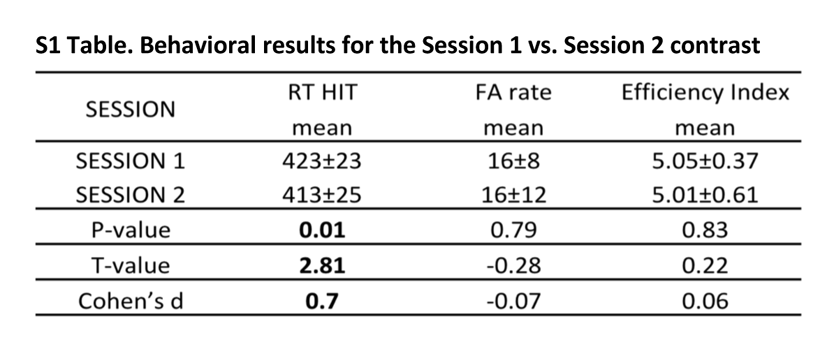

Supplement: S1 Table — Response time to Go condition (RT HIT), false alarm rate (FA) and efficiency index in session 1 and session 2. We report the means ± standard deviation, p- and t-values of the comparison (paired t-test) and Cohen’s d effect size. (TIF) [file pone.0194936.s004.tif]

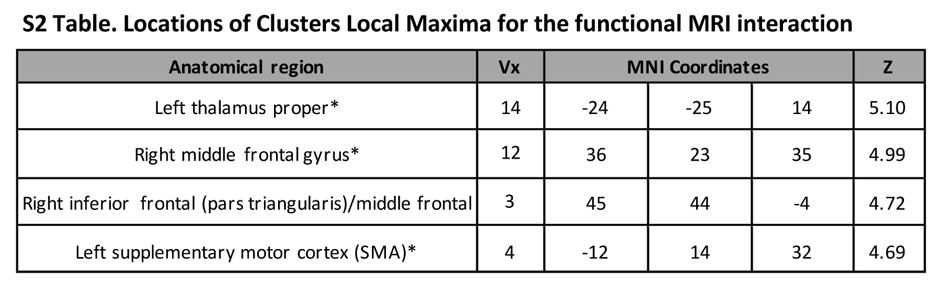

Supplement: S2 Table — Stereotaxic brain MNI coordinates for peak-voxels of the functional interaction (NoGo-Go)sham>(NoGo-Go)stim. Results are represented at pFWE< 0.05 corrected at voxel level (min cluster size = 3). An asterisk (*) indicates the anatomical position is retrieved from the nearest GM for this particular peak. Vx = voxel size, Z = Z-score of the peak. (TIF) [file pone.0194936.s005.tif]
